# The Role of Health Belief Model Constructs and Content Creator Characteristics in Social Media Engagement: Insights from COVID-19 Vaccine Tweets

**DOI:** 10.3390/healthcare12181845

**Published:** 2024-09-14

**Authors:** Xiaofeng Jia, Soyeon Ahn, Michelle I. Seelig, Susan E. Morgan

**Affiliations:** 1 School of Media & Communication, Bowling Green State University, Bowling Green, OH 43403, USA; 2 Department of Educational and Psychological Studies, School of Education and Human Development, University of Miami, Coral Gables, FL 33146, USA; s.ahn@miami.edu; 3 Department of Interactive Media School of Communication, School of Communication, Coral Gables, FL 33146, USA; mseelig@miami.edu; 4 Department of Communication Studies, School of Communication, University of Miami, Coral Gables, FL 33146, USA; semorgan@miami.edu

**Keywords:** COVID-19 vaccine, social media engagement, health belief model, content creator characteristics, content analysis

## Abstract

Introduction: The Health Belief Model (HBM) has been widely studied, but it is unclear how social media post creators use HBM constructs to influence the public’s awareness of health topics, particularly for COVID-19 preventative health behaviors. Moreover, there is limited knowledge about how content creators enhance user engagement with COVID-19 vaccine tweets. Methods: A content analysis of COVID-19 vaccine tweets (*n* = 362) examined how HBM constructs were used in social media posts and their relationship to content creator characteristics (e.g., race, education level) and user engagement behaviors (e.g., number of favorites). Findings: Content creators’ tweets about COVID-19-related topics generally centered on two HBM constructs—benefits and barriers—while fewer tweets emphasized perceived susceptibility or self-efficacy; tweets containing these constructs were retweeted less often. Findings revealed that tweets from politicians, health experts, and white and Asian sources emphasized the perceived benefits of the COVID-19 vaccine. Individual tweets, especially from Black individuals and celebrities, generated more user engagement. Conclusions: Understanding the factors that contribute to social media user engagement with health-related content is important for designing more focused and impactful health communication campaigns and promoting healthier habits and perspectives. Thus, by demonstrating the possible relevance of the HBM to digital communication strategies or health campaigns, our study provides useful guidance for health promoters and policymakers who use social media to raise public health awareness.

## 1. Introduction

Social media has emerged as a crucial tool for disseminating health information and influencing public health behaviors, especially during the COVID-19 pandemic [1,2]. As lockdowns and social distancing measures isolated individuals physically, social media platforms like Twitter, Facebook, and Instagram became essential spaces for staying informed and connected with others [3,4]. The rapid spread of information on social media platforms significantly impacted COVID-related public health responses and behaviors worldwide [5,6]. For instance, COVID-19 vaccine opposition on Twitter, which increased by 80% in 4 months (October 2019 to February 2020), centered on themes such as negative health impact, vaccine ingredients, and vaccine safety [7]. Social media has clearly influenced health behaviors related to vaccine acceptance, but it has also served as a channel for enhancing vaccine resistance [8].

Social media may have had more impact than traditional media on COVID-related health behaviors because these platforms allow users to interact directly with the content they choose to view [9]. Disseminating messages via social media can more effectively foster healthy behaviors by using frameworks for health behavior like the Health Belief Model (HBM) [10]. The HBM posits that individuals’ health behaviors are influenced by their perceptions of susceptibility to a health threat, the severity of its consequences, the benefits of taking action, and the barriers to doing so, along with cues to action and self-efficacy [11]. Although the HBM is a widely used framework for studying health-related behavior, its application to social media posts is still an open area of inquiry. Previous studies have shown the predictive value of HBM in a digital environment [12,13], but few have investigated how health-related social media posts use HBM constructs to influence vaccination intention. Moreover, there is a lack of research on how demographic characteristics of content creators (e.g., race, gender, and professional background) use HBM constructs in their health-related social media posts and how the specific relationship between utilizing different HBM constructs—such as perceived susceptibility, severity, benefits, barriers, and self-efficacy—impacts user engagement behaviors.

The current study focuses on how HBM has been employed to help create effective and appealing social media messages [14,15]. Specifically, we employ content analytic strategies to systematically analyze the use of HBM constructs in health-related tweets and the demographic characteristics of content creators, and examine how content creator characteristics influence user engagement. By doing so, we aim to identify how these factors influence user engagement can guide the development of more targeted and effective communication strategies or campaigns on social media and how this can support health communicators and policymakers in their efforts to tackle vaccine hesitancy and propel public health initiatives forward [16,17].

### 1.1. HBM Construct and Social Media Messages

The Health Belief Model (HBM) is a psychological framework useful for explaining and predicting health-related behaviors, particularly those related to the uptake of health behaviors [10]. According to HBM, individuals’ health behaviors are influenced by their personal beliefs about health conditions and the perceived benefits and barriers to taking action [10,18]. HBM consists of several core constructs: perceived susceptibility, perceived severity, perceived benefits, perceived barriers, cues to action, and self-efficacy [10,19]. Perceived susceptibility refers to an individual’s belief about the risk of acquiring a disease or health condition, while perceived severity pertains to the belief in the seriousness of contracting an illness and its potential consequences. Perceived benefits involve an individual’s assessment of the positive outcomes of a health behavior, whereas perceived barriers denote the obstacles to performing the behavior. Self-efficacy reflects confidence in one’s ability to perform the behavior successfully [11].

The HBM has been widely used to help design interventions promoting health behavior change. Several meta-analyses and systematic reviews have demonstrated the HBM’s effectiveness in predicting preventive health behaviors [20,21,22,23,24]. Specifically, the HBM has been one of the most common theoretical frameworks used to explain vaccine hesitancy in various settings and populations and has been used to design interventions to promote vaccination behaviors (e.g., seasonal influenza vaccination [25]; H1N1 vaccination [26]; Polio vaccination [27]; HPV vaccination [28]).

The HBM is a useful tool for health communication on social media [29], where it has been used for various health topics, such as COVID-19 prevention [12,15], Zika virus [14], and healthy eating habits [13]. The HBM can help address how people perceive different factors influencing their actions. However, individual HBM constructs have different impacts depending on the health issue. For example, Alhaimer (2022) found that cues to action and perceived benefits influenced COVID-19 prevention messages disseminated by government agencies on social media [12]. Zhang and colleagues (2017) found that HBM constructs were seldom used, but they increased social media engagement when used [13]. More research is needed to understand which HBM elements work best in social media messages that make them more engaging and thus relate to preventative health behaviors.

### 1.2. Social Media User Engagement

User engagement is characterized as a dimension of user experience encompassing a state of cognitive and emotional involvement [30], such as searching, viewing, commenting on, and sharing content [31]. Many studies use quantitative metrics, such as the number of likes, shares, comments, views, followers, or clicks, to gauge both the level and the valence of engagement. Social media user engagement with posts is correlated it with users’ offline actions, such as buying products or services [32,33,34]. Previous research has measured the effects of social media user engagement in a variety of ways. For example, the outcome of Voorveld et al.’s (2018) research shows that engagement on different social media platforms is associated with unique experiences, and these experiences are related to users’ evaluations of social media sources [34]. Therefore, it is important to understand the relationship between the characteristics of social media posts and engagement behaviors.

Prior research demonstrates that content creators’ characteristics, such as their race, gender, professional background, or number of followers, impact the level of engagement they receive on social media [35]. For example, researchers have shown that a creator’s age and gender play the most significant role in user engagement [36,37]. Other studies found that creators’ race is associated with user engagement. For example, one study concluded that Black individuals’ tweets about vaccines received more engagement because of public concern about the ways COVID-19 affected Black communities [38].

In addition to demographic factors such as gender and race, influential individuals, including celebrities, politicians, and public figures, receive significantly greater user engagement when they post on social media [39]. Research has demonstrated that celebrities tend to receive a notably higher number of favorites for their social media posts [40,41,42]. Similarly, evidence indicates that politicians who adopt a celebrity-like approach on social media platforms garner substantial user engagement [43,44]. Furthermore, research has revealed a positive association between the content creator’s number of followers and user engagement by those followers [45,46].

### 1.3. The Present Study

**Figure 1:**
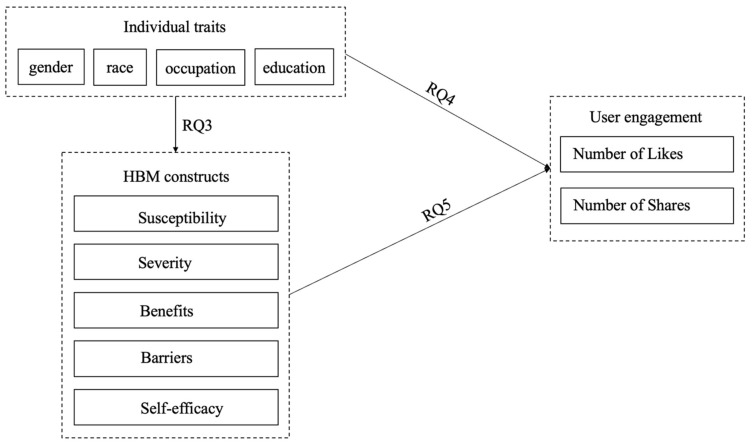
The conceptual model.

Through a content analysis of the COVID-19 vaccine social media posts from 5 January 2021 to 13 January 2022, we aimed to explore the association between content creators’ traits and the use of HBM constructs. Our goal was to investigate how content creators’ demographic traits and HBM constructs in social media posts affect user engagement, measured by the number of retweets and likes. In the course of our study, we identified which specific characteristics of content creators are related to the use of HBM constructs in their social media posts. A primary goal of the study was to advance understanding of the ways in which content creators influence audiences to adopt health behaviors through specific message strategies disseminated through social media. Accordingly, this study explored the following research questions, as illustrated in Figure 1:

- RQ1: Which constructs of the HBM are evident in COVID-19 vaccine tweets?
- RQ2: What are the demographic characteristics of the Twitter content creators responsible for generating tweets about the COVID-19 vaccine?
- RQ3: Do the demographic characteristics of Twitter content creators use specific HBM constructs in their tweets?
- RQ4: Are there specific demographic characteristics associated with Twitter content creators that result in higher user engagement?
- RQ5: What HBM constructs are typically used in tweets that lead to higher user engagement?

## 2. Methods

We conducted a quantitative content analysis on COVID-19 vaccine tweets from 5 January 2021 to 13 January 2022. Quantitative content analysis is a method for measuring the occurrences of words, themes, or concepts in message content [47]. This method involves developing a coding scheme based on relevant literature, applying it to a sample of content, and ensuring intercoder reliability through statistical measures [48]. The coded data are then analyzed to identify patterns, trends, and relationships, offering a replicable approach to understanding content [49]. Each social media post was a unit of analysis.

### 2.1. Sample

A sample of tweets was generated from the CoVaxxy dataset [50], which has public English tweets about COVID-19 vaccines generated from 4 January 2021 to 20 January 2022, using Twitter’s Application Programming Interface (API). The CoVaxxy dataset has tweets for each day and is stored in sub-datasets that differ by posting dates. Since the CoVaxxy dataset is substantially large (*n* = 308,577,272 tweets), a purposive sample of tweets was extracted based on the following criteria:

1. Selection Based on Significant Events: To ensure the sample was relevant to significant COVID-19 vaccine events, at least one significant event related to the COVID-19 vaccine (e.g., CDC recommends pausing the J&J vaccine) had to have occurred on that date.
2. Exclusion of Retweets: Our study focused on the characteristics of tweets and creators leading to user engagement. Therefore, we included only original tweets and excluded retweets.
3. High Engagement Tweets: Our sample prioritized tweets with high user engagement that consisted of the top 1000 tweets with the most retweets and the top 1000 tweets with the most favorites.
4. Bot Exclusion: To filter out bot-generated tweets, Botometer was used, a tool that assigns a Complete Automation Probability (CAP) score to each Twitter account. Accounts with a CAP score above 95% were labeled as bots.

**Figure 2:**
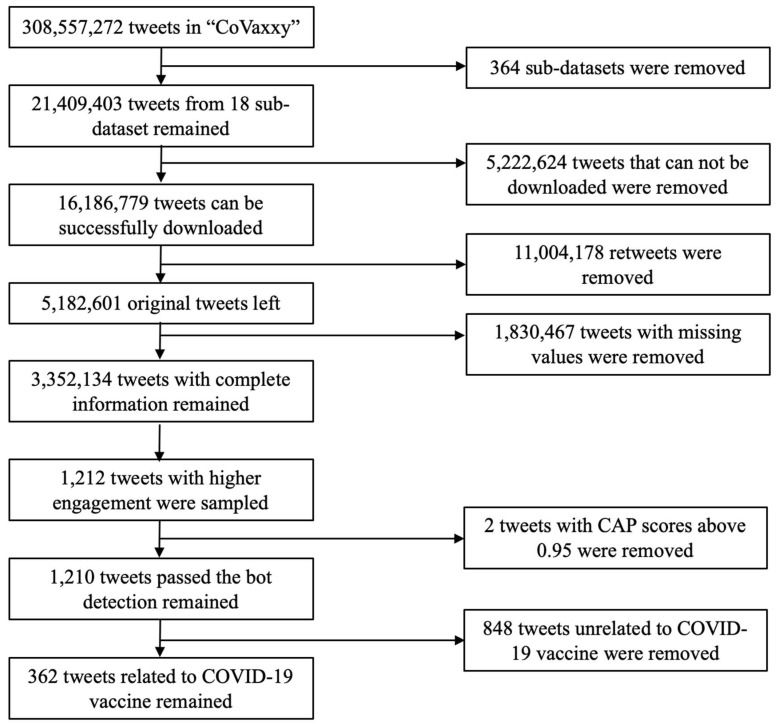
The flow chart of the data collection process.

After applying the extraction criteria to the CoVaxxy dataset, 1210 tweets authored by 699 distinct tweeters were compiled. Next, tweets were coded by two coders to determine if they used the constructs of the HBM; we found that 362 of 1210 tweets posted by 277 tweeters were about the COVID-19 vaccine and contained HBM constructs. Figure 2 illustrates the data collection process.

### 2.2. Coding Scheme

We coded each tweet as a categorical variable that measures each HBM construct, using 1 (present) or 0 (absent). The coding scheme was developed based on the HBM and adapted from research related to the COVID-19 vaccine [51,52,53,54]. Recording units included five items representing perceived susceptibility (e.g., high death rate, COVID-19 can be fatal), five items targeting perceived severity (e.g., children, elderly, pregnant women), six items depicting the perceived benefits of COVID-19 vaccination (e.g., reduced chance of infection, relief from worrying), seven items representing perceived barriers to vaccination (e.g., side effects, inconvenience), and three items targeting perceived self-efficacy (e.g., COVID-19 vaccines are free, convenience of getting the vaccine).

To ensure all items were relevant for evaluating tweets related to the COVID-19 vaccine, we randomly selected 1000 tweets from the ‘CoVaxxy’ dataset, in addition to the 1210 tweets already in our dataset, and coded them using the items. Following the process, we eliminated the items that had no relevance for measuring the HBM construct and included some sub-items that were frequently used to assess HBM constructs and source attributes. For example, we added “social isolation/mental health issues” for the perceived severity of COVID-19 and “vaccination passport/mandatory vaccine requirement” for perceived barriers to the COVID-19 vaccine. Subsequently, we had a total of 44 items, including 8 for susceptibility, 9 for severity, 9 for the benefits of COVID-19 vaccination, 15 for barriers to vaccination, and 3 items for self-efficacy.

The coding sheet for creators’ characteristics consisted of self-disclosed information on their Twitter profile: creator (e.g., organization or an individual); gender (e.g., female, male, other, or absent); race/ethnicity (e.g., white, Black, Asian or Pacific Islander, American Indian or Alaskan Native, Hispanic, other, or absent); occupation (e.g., health professional, public health expert, celebrity, politician, other, or absent); education (e.g., MD, PhD, Master’s degree, other, or absent). If there was no self-disclosure, two coders independently coded the person they perceived from their profile; for example, if a content creator’s portrait was of a white male, the coder coded their race as white and their gender as male. The items were coded as missing values if these characteristics could not be identified. The final codebook is presented in Table 1.

### 2.3. Coding Procedures

To ensure that the coders reached an acceptable level of agreement, both coders underwent comprehensive training sessions, where they familiarized themselves with the coding scheme and practiced coding on a subset of tweets not included in the final analysis. This training helped establish a shared understanding of the coding categories and reduced potential ambiguities. Following the training, they both coded 121 tweets, surpassing the 10% recommended minimum sample size for calculating intercoder reliability [47]. Intercoder reliability was calculated using Cohen’s kappa because all variables were categorical. The average Cohen’s kappa was 0.864 (ranging from 0.74 to 1), indicating acceptable reliability [47]. Next, two coders independently coded the remaining tweets. In cases where discrepancies occurred, the coders engaged in discussions to reach a consensus, ensuring the final coding was agreed upon by all parties. Table 1 presents the intercoder reliability coefficients for all the coding categories.

### 2.4. Data Analysis

To perform analyses and generate frequency tables, the Statistical Package for Social Sciences (SPSS 26.0) was used [55]. Specifically, a series of independent samples *t*-tests, Analysis of Variance (ANOVA), and regression were conducted to answer five research questions. Each HBM construct comprised multiple items, which were summed as a total score for each construct. In the regression analysis, the number of retweets was regressed on the tweet using severity, susceptibility, benefits, barriers, and self-efficacy as predictors.

## 3. Results

### 3.1. Descriptive Statistics

The average number of words in these tweets ranged from 5 to 55 (*M* = 33.27, *SD* = 12.59). The number of retweets ranged from 34 to 112,841 (*M* = 3778.28, *SD* = 7886.67), and favorites ranged from 1836 to 695,439 (*M* = 19,426.58, *SD* = 45,323.17). The descriptive statistics of posts, including the HBM constructs and creators’ characteristics, are shown in Table 1.

### 3.2. Characteristics of COVID-19 Vaccine-Related Posts

Research Question 1 sought to determine which constructs of the HBM are evident in COVID-19 vaccine tweets. As indicated in Table 2, the most frequently tweeted HBM constructs were perceived barriers to getting the COVID-19 vaccine (*M* = 0.88, *SD* = 0.70), followed by tweets that highlighted perceived benefits (*M* = 0.63, *SD* = 0.93), perceived severity (*M* = 0.26, *SD* = 0.69), self-efficacy (*M* = 0.08, *SD* = 0.30), and perceived susceptivity (*M* = 0.03, *SD* = 0.22).

Research Question 2 examined the demographic characteristics of the content creators responsible for generating tweets about the COVID-19 vaccine. In coding tweets, we found that Twitter content creators were from various countries (e.g., United States, United Kingdom, India, Mexico, etc.). The number of followers for tweeters of these tweets varied from 122 to 80,911,439 (*M* = 2,473,188.10, *SD* = 9,312,872.58). The majority of tweets were created by individuals (*n* = 321, 88.7%), and the remainder were tweeted by organizations (*n* = 41, 11.3%), such as the White House, the Food and Drug Administration (FDA), media, universities, etc. More than half of the tweets were posted by males (*n* = 193, 53.3%), followed by females (*n* = 111, 30.7%) and unspecified (n = 58, 16%). Most posts originated from white people (*n* = 208, 57.5%), fewer originated from Black tweeters (*n* = 35, 9.7%) or from Asian or Pacific Islander tweeters (*n* = 24, 6.6%), and 26.2% (*n* = 95) could not be determined.

### 3.3. Characteristics of Content Creators and User Engagement

To address Research Question 3, we compared the mean values of user engagement to explore the relationship between the characteristics of social media content creators and user engagement levels. First, tweets from individual tweeters received more favorites than those created by organizations (*t* (360) = −2.67, *p* = 0.01). Next, we found a significant difference in the number of retweets (*F* (3, 358) = 3.06, *p* = 0.03) or the number of favorites (*F* (3, 358) = 5.83, *p* < 0.001) depending on the content creator’s race. Specifically, content tweeted by a Black creator was retweeted more than that from white tweeters. Also, tweets created by a Black Twitter user received more favorites than those from white or Asian users. Furthermore, a significant difference was found in the number of favorites a tweet received depending on a tweeter’s occupation (*F* (5, 356) = 2.40, *p* = 0.04). Specifically, tweets created by celebrities received more favorites than those created by health professionals and public health experts. Lastly, there was a significant difference in the number of favorites a tweet received by a tweeter’s education level (*F* (2, 359) = 3.42, *p* = 0.03). Specifically, tweets from people who did not expose their education information received significantly more favorites than those created by people who identified themselves as MD (for the results of the independent samples t-test or Analysis of Variance, please refer to the Supplementary Materials).

### 3.4. Characteristics of Content Creators and Use of HBM in Social Media Posts

Research Question 4 addressed whether the use of HBM constructs in messages differed depending on the traits of Twitter content creators. Significant differences were found for perceived benefits of the vaccine by the creator’s race (*F* (3,358) = 4.46, *p* < 0.01) and the creator’s occupation (*F* (5,356) = 4.30, *p* < 0.01) as well as perceived susceptibility in the tweets by the creator’s occupation of the source (*F* (5,356) = 5.32, *p* < 0.01). Specifically, Black creators did not tweet as much about perceived benefits as white or Asian creators. Likewise, creators who were lawyers, scholars, and businesspeople tweeted less about perceived benefits. Creators with these occupations also tweeted more on perceived susceptibility than celebrities, politicians, or media practitioners. While politicians tweeted more about the benefits of taking the COVID-19 vaccine than other occupations.

### 3.5. Relationship between HBM Use in Social Media Posts and User Engagement

With regard to our last research question regarding what HBM constructs are typically used in tweets that lead to higher user engagement, as shown in Table 3, results from a multiple regression analysis indicate that tweets emphasizing perceptions of susceptibility (*b* = −0.14, *p* = 0.01) or perceived self-efficacy (*b* = −0.13, *p* = 0.01) are less likely to be retweeted by social media users. Moreover, none of the HBM constructs significantly predicted the number of favorites that a tweet received.

## 4. Discussion

The current study used quantitative content analysis to explore the types of persuasive appeals, as articulated by the Health Belief Model, that are used by content creators in their tweets about COVID-19. Additionally, we were interested in how user engagement may be influenced by the demographic characteristics of content creators. Finally, we explored how the number of HBM constructs in tweets relates to specific characteristics of content creators.

Our analyses revealed that the most frequently used HBM constructs were the perceived benefits and perceived barriers to receiving the COVID-19 vaccine, while HBM constructs like self-efficacy were employed far less often. We discovered that tweets targeting perceived severity, susceptibility, benefits, barriers, and self-efficacy tended to receive more retweets. However, tweets emphasizing perceived susceptibility or self-efficacy were less likely to be retweeted. Perceived susceptibility might not be as compelling or might be perceived as less relevant if the audience does not see themselves at risk. Additionally, self-efficacy messages, which emphasize an individual’s ability to act, might not generate as much public interest or urgency compared to messages about severity or benefits.

Regarding Twitter posts about COVID-19, most were by men, white individuals, health professionals, politicians, and media practitioners. This demographic breakdown suggests a potential imbalance in whose voices are most prominent in the public discourse about COVID-19 on Twitter. Previous research has shown that men and individuals from higher socioeconomic backgrounds, which are often correlated with being white in many contexts, are more likely to have higher social media activity and greater access to digital technologies [56]. This can lead to their voices being disproportionately represented in online discussions, including those about health topics like COVID-19.

Interestingly, the HBM constructs that social media content creators used in their posts appear to be related to some of their demographic characteristics. Specifically, white or Asian creators focused more on the benefits of the COVID-19 vaccine than those who were Black. Previous research has shown that different racial and ethnic groups may perceive health risks and benefits differently due to historical, social, and economic contexts [57]. For example, Black communities in the United States have historically faced medical mistreatment and systemic racism, leading to higher levels of medical distrust and vaccine hesitancy [58]. As a result, Black content creators might focus less on benefits and more on addressing barriers or concerns related to the vaccine. Moreover, there were significant differences based on users’ occupations highlighting perceived benefits in their posts, finding that politicians were more likely to mention the benefits of vaccination in their tweets than others. Politicians often play a crucial role in public health advocacy and policy implementation. Highlighting the benefits of vaccination can be seen as an effort to encourage public compliance and support for vaccination campaigns, aligning with their roles in promoting public health measures.

Our analyses showed other distinct patterns associated with source characteristics. Tweets about the COVID-19 vaccine from individuals received more favorites than those from organizations. The higher engagement with tweets from individuals compared to organizations suggests that personal narratives and voices may be more relatable and engaging for the public on social media, which aligns with the theory of parasocial interaction [59]. This finding indicates that public health communication strategies on social media might benefit from leveraging personal stories and individual endorsements to enhance engagement.

There were also patterns associated with the race of the source. The greater number of retweets and favorites for tweets from Black sources compared to white and Asian sources highlights the importance of racial representation in health communication. This finding is consistent with studies showing that messages from individuals within one’s racial or ethnic group can enhance trust and relatability [60]. It underscores the need for diverse voices in public health campaigns to reach and resonate with broader audiences, particularly marginalized communities.

Celebrity status may also matter for engagement with tweets about COVID-19. Tweets from celebrities received more favorites than those created by health professionals or public health experts, but content creators who identified themselves as MD or Ph.D. received fewer favorites than those who did not share their education information. The lower engagement with tweets from MD or Ph.D. holders who disclosed their educational background suggests a possible perception of these messages as less relatable or overly technical. Previous research indicates that while expertise is valued, it must be communicated in an accessible and engaging manner to be effective [61].

### 4.1. Implications

This research offers valuable insights with both theoretical and practical implications. First, this research advances our understanding of how the HBM can serve as a robust theoretical framework for designing and enhancing social media messages and interventions [14,16]. In line with earlier studies, our findings indicate that HBM is a useful behavioral model for interpreting health-related posts on social media [15]. Furthermore, our study demonstrates how each HBM concept works in real-time scenarios. For instance, our results indicate that the severity and susceptibility constructs are not frequently utilized in tweets about getting the COVID-19 vaccine. These insights aid researchers and practitioners in comprehending how social media users express themselves regarding health issues, such as the COVID-19 pandemic, and specifically, what information strategies they employ [16]. We believe that this finding will guide health professionals, public health policymakers, and social media influencers to craft effective social media messages, thereby enhancing the likelihood of achieving desired outcomes. On the other hand, it is equally important to emphasize the reliability of their content to ensure that information shared is accurate and trustworthy.

Additionally, this research adds knowledge to the existing literature regarding the relationship between messaging strategies used to promote health behaviors and user engagement from the perspective of the HBM. Prior studies have employed the HBM to examine the content and engagement with vaccine-promoting posts on social media [62]. Our results demonstrate that social media posts containing threat content receive relatively low user engagement. This suggests that when sources emphasize the severity of and susceptibility to a disease, audiences are less likely to engage with such posts, perhaps because these posts do not also emphasize strategies to avoid the threat, which may demoralize users. Therefore, to boost user engagement, social media content creators should consider modifying messages about the severity and susceptibility of health-related information to also include efficacy components.

Lastly, our results align with prior research showing that creator characteristics, such as race, gender, or professional background, influence engagement levels in social media contexts [35]. For example, we found that race is associated with user engagement, particularly with Black sources. Chandler et al. (2021) demonstrated that higher engagement with Black sources’ vaccine-related tweets indicates a heightened interest in or concern about COVID-19’s impact on Black communities [38]. This finding suggests that social media can amplify the voices of marginalized communities and foster greater understanding and empathy among diverse racial and ethnic groups [63]. Particularly for the Black community, social media holds the potential to effectively convey crucial information about health issues that are not well understood. If appropriately disseminated, this information can encourage positive health behaviors and ultimately help reduce health disparities.

### 4.2. Limitations and Future Research

Given the unique nature of our sample, it is important to consider potential limitations when interpreting the findings of this study. First, data were collected from CoVaxxy, a public dataset of COVID-19 vaccine-related tweets from 4 January 2021 to 20 January 2022, totaling 308,577,272 tweets. Due to the size of the dataset, only the top 1210 tweets by number of favorites and retweets were coded, with 362 vaccine-related tweets forming the final sample. Additionally, our analysis was limited to English-language Tweets, which may not capture the full global context and perspectives of the pandemic. Moreover, we focused on the tweets that received more favorites and retweets, potentially limiting the generalizability of our findings to only the most popular content. These limitations prevent a truly comprehensive understanding of COVID-19 vaccine discussions on Twitter. However, this study developed a coding scheme for identifying common messaging strategies and content creator characteristics, which can be used in future research to train machine learning (ML) techniques for broader data analysis.

The results showed the relationship between the characteristics of content creators, such as occupation, education level, and user engagement. However, these relationships could be confounded by factors such as the number of followers of content creators. In the present study, the number of favorites, retweets, and followers is highly skewed. This poses a challenge in controlling the impact of the number of followers on the association between content creators’ traits and user engagement. In future studies, it would be beneficial to balance the sampling of content creators based on the number of followers, in order to mitigate the confounding effects of the number of followers.

Moreover, the conclusions related to Black individuals should be interpreted with caution due to their relatively small representation in the study sample. However, this limitation highlights the importance of focusing on how minority and marginalized groups discuss the COVID-19 vaccine on social media. Future research should prioritize these groups, exploring how race intersects with factors such as geographical location and age to provide a more nuanced understanding of how these variables collectively influence social media engagement and public responses.

Furthermore, except for the metadata that were extracted from Twitter, the HBM constructs in the social media posts and content creators’ personal traits were based on coders’ perceptions or users’ self-disclosure on social media rather than information provided by sources themselves. Thus, our assessments of some source characteristics may not be entirely accurate. Coders’ perceptions can nonetheless be justified because the correlation between content creators’ attributes and user engagement behaviors in the real world hinges on the audience’s perception of their personal traits. In this study, coders were systematically trained, and all variables reached an acceptable intercoder reliability, which we believe further supports the validity of our procedures. There is potential to address this issue in future research by conducting strictly controlled experiments to further explore the causal relationship between content creators’ characteristics and audience engagement behaviors.

This study focused on engagement behaviors as a key outcome, particularly favoriting and retweeting. However, future research should explore which posts receive the most comments, as well as the valence and content of these comments. Future research should focus on the motivations for each type of engagement behavior, and the characteristics of the audience who exhibit the greatest or least likelihood of engagement with different types of social media posts about vaccines or other health behaviors. This includes examining whether liking, sharing, and commenting reflect personal attitudes or anticipation of followers’ appreciation and whether these behaviors indicate actual agreement with the content. Future research should address the limitations of this study by exploring a broader dataset, controlling for the number of followers, and conducting controlled experiments to understand better the causal relationships between content creator traits and user engagement.

Lastly, based on the results of this research, future research could focus on developing mathematical models to predict user engagement based on content creator characteristics, such as race or profession, adding a quantitative and predictive dimension to the analysis of social media trends across various health topics. This could involve using regression or machine learning techniques to estimate engagement levels based on key variables, thereby enhancing the predictive power of our analysis and validating findings with real-time data. Additionally, future studies could explore the correlation between social media engagement and real-world public health outcomes, such as vaccine uptake rates or infection trends, to better understand how communication efforts on platforms like Twitter influence actual behavioral outcomes.

## 5. Conclusions

In sum, this study provides valuable information concerning the detailed characteristics of tweets related to the COVID-19 vaccine on Twitter, as well as the relationship between the use of HBM constructs, creator characteristics, and user engagement of these posts. As evident here, perceived benefits and perceived barriers are the most frequently used HBM constructs in COVID-19-related posts. In addition, tweets from Black creators and celebrities garnered higher engagement, emphasizing the importance of creator characteristics in influencing user engagement. The differential engagement levels identified in this study underscore the importance of leveraging relatable and authoritative voices to enhance the reach and effectiveness of health messages. These insights are valuable for health professionals, policymakers, and influencers aiming to craft effective health messages that resonate with diverse audiences. By emphasizing relevant HBM constructs in social media messages and collaborating with influential individuals, health communicators can more effectively engage diverse audiences and promote public health initiatives. Understanding the dynamics of user engagement in the context of COVID-19 can provide a roadmap for crafting compelling and impactful health communications.

## Figures and Tables

**Table 1:** Coding sheet, intercoder reliability, and descriptive statistics.

Variables	Sub-Items	Number of Presences/Range	Percent (%) of Presences/M& SD	Cohen’s Kappa
Relevance				0.83
Metadata	Coding_ID			
	Tweet_ID			
	Created_at			
	Language			
	Full_text			
	Word_counts	5–55	*M* = 33.27 *SD* = 12.59	
	User_ID			
	User_name			
	Location			
	User_description			
	Number of followers	122–80,144,312	M = 2,473,188.10	
			SD = 9,312,872.58	
User engagement	Number of likes	1836–695,439	*M = 19,426.58*	
			*SD = 45,323.17*	
	Number of retweets	34–112,841	M = 3778.28	
			SD = 7886.67	
Source characteristics	**Source**			0.91
	1: Organization	41	11.3	
	2: Individual	321	88.7	
	**Gender**			0.91
	0: N/A	58	16	
	1: Female	111	30.7	
	2: Male	193	53.3	
	**Race/ethnicity**			0.88
	0: N/A	95	26.2	
	1: White	208	57.5	
	2: Black	35	9.7	
	3: Asian or Pacific Islander	24	6.6	
	4: American Indian or Alaskan Native	0	0	
	5: Hispanic	0	0	
	6: Other	1	0.3	
	**Occupation**			0.80
	0: N/A	158	43.6	
	1: Health professionals and public health experts	45	12.4	
	2: Celebrities (actors, singers, SMIs)	15	4.1	
	3: Politicians	43	11.9	
	4: Media practitioners (writers, journalists, anchors)	66	18.2	
	5: Others (artists, scholars, businessmen, lawyers)	35	9.7	
	**Education**			0.91
	0: N/A	316	87.3	
	1: MD	35	9.7	
	2: PhD	11	3	
	3: Master’s	0	0	
	4: Others	0	0	
Targeting perceived severity	Item1: Death rate is high	23	6.4	0.89
	Item2: COVID-19 can be fatal	32	8.8	0.85
	Item3: COVID-19 has serious after effects	11	3	1
	Item4: Lost job	0	0	N/A
	Item5: Family/friends dying	5	1.4	1
	Item6: Widespread transmission	22	6.1	0.89
	Item7: Social isolation/mental health issues	1	0.3	N/A
	Item8: Expensive treatment	1	0.3	N/A
Targeting perceived susceptibility	Item1: Elderly people	1	0.3	N/A
	Item2: Disadvantaged groups	1	0.3	N/A
	Item3: Healthcare workers	0	0	N/A
	Item4: Pregnant women	1	0.3	N/A
	Item5: Children	4	1.1	1
	Item6: Unvaccinated people	0	0	N/A
	Item7: People who do not wear masks	1	0.3	N/A
	Item8: Homeless	0	0	N/A
	Item9: People with specific health conditions	4	1.1	1
Targeting perceived benefits of COVID-19 vaccination	Item1: Reduce the chance of infection	67	18.5	0.76
	Item2: Decrease the severity and the chance of having complications	26	7.2	0.83
	Item3: Feel protected from COVID-19 infection	2	0.6	N/A
	Item4: Restore a normal social life	15	4.1	0.80
	Item5: Relief from worrying	0	0	N/A
	Item6: Protect family, friends, and others	36	9.9	0.79
	Item7: Transmission reduction/end the pandemic	58	16	0.91
	Item8: Save medical resources	14	3.9	N/A
	Item9: Works for variants	10	2.8	N/A
Targeting perceived barriers of COVID-19 vaccination	Item1: Efficacy	78	21.5	0.88
	Item2: Safety	112	30.9	0.74
	Item3: Side effects	19	5.2	0.74
	Item4: Getting sick from COVID-19 vaccine	1	0.3	N/A
	Item5: Inconvenience of getting vaccinated	2	0.6	N/A
	Item6: Transportation to vaccination site	1	0.3	N/A
	Item7: Don’t have time to get vaccinated	1	0.3	N/A
	Item8: Conspiracy theory	46	12.7	0.76
	Item9: Cannot accept injection	1	0.3	N/A
	Item10: Lack of knowledge/data	17	4.7	0.85
	Item11: Rushed	9	2.5	N/A
	Item12: Vaccination passport/mandatory vaccine requirement	22	6.1	0.79
	Item13: Family/friends do not support/social norm	3	0.8	N/A
	Item14: Misinformation/ disinformation	5	1.4	N/A
	Item15: History of medical exploitation (Black people)	1	0.3	N/A
Targeting self-efficacy	Item1: Getting vaccinated is easy	12	3.3	N/A
	Item2: Getting vaccinated is free	10	2.8	1
	Item3: Have ability to deal with side effects	6	1.7	N/A

Note. N/A: no data were available to calculate the intercoder reliability.

**Table 2:** Descriptive statistics of HBM constructs.

Constructs	*Min*	*Max*	*M*	*SD*
Severity	0	3	0.26	0.69
Susceptivity	0	3	0.03	0.22
Benefits	0	4	0.63	0.93
Barriers	0	3	0.88	0.7
Self-esteem	0	2	0.08	0.3

**Table 3:** Use of HBM in messages and user engagement.

	*b*	*SE*	*β*	*Back-Transformed* *β*	*t*	*p*
Intercept	7.73	0.11			70.16	<0.01 **
Severity	0.11	0.08	0.08	1.08	1.42	0.16
Benefits	−0.04	0.06	−0.04	0.96	−0.64	0.52
Barriers	0.005	0.08	0.004	1.00	0.06	0.95
Susceptibility	−0.81	0.31	−0.14	0.87	−2.59	0.01 *
Self-efficacy	−0.50	0.20	−0.13	0.88	−2.48	0.014 *

Note. Due to non-normality, the log-transformed dependent variable was used as a dependent variable in the regression. Therefore, back-transformed standardized coefficients are present in the table. * *p* < 0.05, ** *p* < 0.01.

## Data Availability

The original contributions presented in the study are included in the article; further inquiries can be directed to the corresponding author.

## Supplementary Materials

The following supporting information can be downloaded at: https://www.mdpi.com/article/10.3390/healthcare12181845/s1, Table S1. Characteristics of content creators and user engagement. Table S2. Characteristics of content creators and use of HBM in messages.
