# Supplementary material for: The Role of Health Belief Model Constructs and Content Creator Characteristics in Social Media Engagement: Insights from COVID-19 Vaccine Tweets
Source: Healthcare (Basel). 2024 Sep 14;12(18):1845. doi: 10.3390/healthcare12181845 (PMC11431524; doi:10.3390/healthcare12181845)
Supplement: Supplementary file 1 [file healthcare-12-01845-s001.zip › healthcare-3192161-supplementary.pdf]

Supplemental Materials

Supplemental Materials Table S1. Characteristics of content creators and user engagement

Variable	Group	*n*	*M*	*SD*	*t/F*	*p*
	**Retweets**					
**Source**	individuals	321	3761.36	7855.76	-0.61	0.54
	organizations	41	3910.73	8223.59		
**Gender**	female	111	3199.90	3720.63	-1.74	0.08
	male	193	4215.18	9696.97		
**Race**	white	208	3028.69	3207.73	3.06	**0.03***
	black	35	7119.06	11320.69		
	Asian	24	2703.00	2800.12		
**Occupation**	health professionals & public health experts	45	2607.58	1887.97	0.60	0.70
	celebrities	15	3873.47	6017.38		
	politicians	43	2996.67	3212.58		
	media practitioners	66	4303.48	8357.01		
	others	35	3315.71	3560.26		
**Education**	MD	35	2549.40	1528.41	0.37	0.69
	PhD	11	4428.91	3751.63		
	others	316	3891.74	8389.77		
	**Favorites**					
**Source**	individuals	321	11072.63	14029.36	2.67	**0.01***
	organizations	41	20493.60	47777.57		
**Gender**	female	111	16478.36	19179.68	-1.02	0.31
	male	193	23756.28	59656.03		
**Race**	white	208	15920.10	18669.78	5.83	**0.001***
	black	35	38762.34	64778.42		
	Asian	24	14737.25	18363.20		
**Occupation**	health professionals & public health experts	45	9224.71	9092.55	2.4	**0.04***
	celebrities	15	28138.20	34944.94		
	politicians	43	16011.98	14733.41		
	media practitioners	66	22678.23	47858.67		
	others	35	17661.26	20707.93		
**Education**	MD	35	9347.60	9935.12	3.42	**0.03***
	PhD	11	16763.64	12733.44		
	others	316	20635.62	48222.03		

Supplemental Materials Table S2. Characteristics of content creators and use of HBM in messages

Variable	Group	*n*	*M*	*SD*	*t/F*	*p*
**Targeting Severity**						
**Source**	**individuals**	**321**	**0.28**	**0.72**	**1.93**	**0.06**
	organizations	41	0.12	0.46		
Gender	female	111	0.32	0.80	0.69	0.49
	male	193	0.26	0.69		
Race	white	208	0.30	0.68	0.58	0.63
	black	35	0.23	0.72		
	Asian	24	0.13	0.73		
Occupation	health professionals & public health experts	45	0.40	0.94	1.60	0.16
	celebrities	15	0.53	1.06		
	politicians	43	0.40	0.73		
	media practitioners	66	0.20	0.61		
	others	35	0.26	0.56		
**Education**	**MD**	**35**	**0.31**	**0.83**	**1.82**	**0.16**
	PhD	11	0.64	1.21		
	others	316	0.24	0.65		
**Targeting Susceptibility**						
**Source**	**individuals**	**321**	**0.04**	**0.23**	**2.86**	**0.01**
	organizations	41	0.00	0.00		
Gender	female	111	0.05	0.33	0.80	0.42
	male	193	0.03	0.17		
Race	white	208	0.25	0.02	0.02	0.99
	black	35	0.17	0.03		
	Asian	24	0.20	0.04		
Occupation	health professionals & public health experts	45	0.07	0.25	**5.32**	**<.001**
	celebrities	15	0.00	0.00		
	politicians	43	0.00	0.00		
	media practitioners	66	0.02	0.02		
	others	35	0.20	0.10		
Education	MD	35	0.06	0.24		
	PhD	11	0.09	0.30		
	others	316	0.03	0.22		
**Perceived Benefits**						
Source	individuals	321	0.61	0.93	-1.28	0.20
	organizations	41	0.80	0.95		
Gender	female	111	0.96	0.09	-0.26	0.80
	male	193	0.93	0.07		
**Race**	**white**	**208**	**0.65**	**0.93**	**4.46**	**0.004**
	black	35	0.11	0.40		
	Asian	24	0.83	0.93		
Occupation	health professionals & public health experts	45	0.80	1.04	**4.30**	**0.001**
	celebrities	15	0.27	0.46		
	politicians	43	1.14	1.04		

	media practitioners	66	0.45	0.79		
	others	35	0.46	0.74		
Education	MD	35	0.83	0.95	0.98	0.38
	PhD	11	0.73	1.27		
	others	316	0.60	0.92		
**Perceived Barriers**						
Source	individuals	321	0.89	0.70	1.18	0.24
	organizations	41	0.76	0.73		
Gender	female	111	0.70	0.07	0.48	0.63
	male	193	0.70	0.05		
Race	white	208	0.90	0.71	2.02	0.11
	black	35	1.09	0.56		
	Asian	24	0.88	0.85		
Occupation	health professionals & public health experts	45	0.87	0.69	1.79	0.11
	celebrities	15	1.07	0.46		
	politicians	43	0.63	0.76		
	media practitioners	66	1.00	0.72		
	others	35	0.94	0.80		
Education	MD	35	0.80	0.63	0.25	0.78
	PhD	11	0.91	0.54		
	others	316	0.89	0.72		
**Perceived Self-efficacy**						
Source	individuals	321	0.08	0.30	0.10	0.92
	organizations	41	0.07	0.26		
Gender	female	111	0.34	0.03	1.27	0.21
	male	193	0.28	0.02		
Race	white	208	0.07	0.29	0.65	0.58
	black	35	0.03	0.17		
	Asian	24	0.13	0.34		
Occupation	health professionals & public health experts	45	0.02	0.15	1.24	0.29
	celebrities	15	0.07	0.26		
	politicians	43	0.14	0.47		
	media practitioners	66	0.05	0.21		
	others	35	0.03	0.17		
Education	MD	35	0.03	0.17	0.96	0.38
	PhD	11	0.00	0.00		
	others	316	0.09	0.31		
